# Oryzalexin S biosynthesis: a cross-stitched disappearing pathway

**DOI:** 10.1007/s42994-022-00092-3

**Published:** 2023-01-19

**Authors:** Le Zhao, Richard Oyagbenro, Yiling Feng, Meimei Xu, Reuben J. Peters

**Affiliations:** Roy J. Carver Department of Biochemistry, Biophysics and Molecular Biology, Iowa State University, Ames, IA 50011 USA

**Keywords:** Biosynthetic gene clusters, Phytoalexins, Diterpenoids, Rice evolution

## Abstract

Rice produces many diterpenoid phytoalexins and, reflecting the importance of these natural products in this important cereal crop plant, its genome contains three biosynthetic gene clusters (*BGCs*) for such metabolism. The chromosome 4 *BGC* (*c4BGC*) is largely associated with momilactone production, in part due to the presence of the initiating *syn*-copalyl diphosphate (CPP) synthase gene (*OsCPS4*). Oryzalexin S is also derived from *syn*-CPP. However, the relevant subsequently acting *syn*-stemarene synthase gene (*OsKSL8*) is not located in the *c4BGC*. Production of oryzalexin S further requires hydroxylation at carbons 2 and 19 (C2 and C19), presumably catalyzed by cytochrome P450 (CYP) monooxygenases. Here it is reported the closely related CYP99A2 and CYP99A3, whose genes are also found in the *c4BGC* catalyze the necessary C19-hydroxylation, while the closely related CYP71Z21 and CYP71Z22, whose genes are found in the recently reported chromosome 7 *BGC* (*c7BGC*), catalyze subsequent hydroxylation at C2α. Thus, oryzalexin S biosynthesis utilizes two distinct *BGCs*, in a pathway cross-stitched together by *OsKSL8*. Notably, in contrast to the widely conserved *c4BGC*, the *c7BGC* is subspecies (ssp.) specific, being prevalent in ssp. japonica and only rarely found in the other major ssp. indica. Moreover, while the closely related *syn*-stemodene synthase *OsKSL11* was originally considered to be distinct from *OsKSL8*, it has now been reported to be a ssp. indica derived allele at the same genetic loci. Intriguingly, more detailed analysis indicates that *OsKSL8(j)* is being replaced by *OsKSL11* (*OsKSL8i*), suggesting introgression from ssp. indica to (sub)tropical japonica, with concurrent disappearance of oryzalexin S production.

**Supplementary Information:**

The online version contains supplementary material available at 10.1007/s42994-022-00092-3.

## Introduction

Rice (*Oryza sativa*) is well known for producing many diterpenoid phytoalexins and has served as model system for investigation of these natural products, which are prevalent in cereal crops more generally (Murphy and Zerbe 2020). One of the early findings from these studies was discovery of biosynthetic gene clusters (*BGCs*) associated with such metabolism, which were among the first to be discovered in plants (Nutzmann et al. 2016). In particular, functional identification of the consecutively acting pairs of cyclases led to realization of the proximity of the relevant genes (Prisic et al. 2004; Wilderman et al. 2004). Moreover, the two identified regions each also contained a number of genes for cytochrome P450 (CYP) monooxygenases, which were later demonstrated to act on the diterpenes generated by the co-clustered cyclases (Swaminathan et al. 2009; Wang et al. 2011, 2012; Wu et al. 2011, 2013; Kitaoka et al. 2015).

The use of two consecutively acting cyclases is characteristic of the labdane-related nature of almost all rice diterpenoids. Their production is initiated by bicyclization of the general diterpenoid precursor (*E,E,E*)-geranylgeranyl diphosphate (GGPP) catalyzed by class II diterpene cyclases, which prototypically produce the eponymous labdadienyl/copalyl diphosphate (CPP) as distinct stereoisomers (Peters 2010). For example, all land plants must produce *ent*-CPP and, hence, contain such a CPP synthase (CPS) for phytohormone biosynthesis. For this purpose, *ent*-CPP must then be further cyclized to the tetracyclic olefin *ent*-kaurene by a class I diterpene synthase. In angiosperms the CPS and kaurene synthase (KS) required for gibberellin phytohormone biosynthesis have often given rise to expanded gene families involved in more specialized metabolism, with the latter generally termed KS-like (KSL) (Zi et al. 2014).

In rice, functional characterization of the *syn*-CPP synthase OsCPS4 and subsequently acting *syn*-pimaradiene synthase OsKSL4 led to discovery of co-localization their genes on chromosome 4 (Wilderman et al. 2004; Xu et al. 2004). Notably, the only other genes in the region encoded the closely related paralogs CYP99A2 and CYP99A3, and pair of short-chain alcohol dehydrogenases, defining a BGC (*c4BGC*). Consistent with RNAi knock-down studies (Shimura et al. 2007), it was then shown that CYP99A3 (Wang et al. 2011), and latter CYP99A2 (Kitaoka et al. 2015), react with *syn*-pimaradiene, both introducing oxygen at carbon-19 (C19), as required for momilactone biosynthesis.

Similarly, functional characterization of the *ent*-cassadiene synthase OsKSL7 and upstream *ent*-CPP synthase OsCPS2 dedicated to more specialized metabolism led to discovery of another *BGC* located on chromosome 2 (Cho et al. 2004; Prisic et al. 2004). This *c2BGC* contains multiple members of the CYP71Z and CYP76M sub-families, with some of the latter surprisingly involved in biosynthesis of the momilactones associated with the *c4BGC*, demonstrating interdependent evolution of these two *BGCs* (Kitaoka et al. 2021; Li et al. 2022). Not surprisingly, the presence of CYP76M sub-family members at the *c2BGC* locus is then conserved throughout the *Oryza* genus (Miyamoto et al. 2016), with *c4BGC orthologs* found even more widely (Wu et al. 2022).

More recently reported was another diterpenoid *BGC* on chromosome 7 (*c7BGC*), encoding *ent*-10-oxodepression production, which seems to be almost entirely restricted to the japonica subspecies (ssp.) and is only rarely found in the other major ssp. (indica) of rice (Zhan et al. 2020). While this report only described the phylogenetically related but functionally distinct CYP71Z2 and CYP71Z21, an independent study found the *c7BGC* also contains genes encoding CYP71Z22, which is closely related and functionally redundant with CYP71Z21, as well as CYP71Z30, albeit this latter is found as a pseudo-gene even in some cultivars (cv.) from ssp. japonica (Liang et al. 2021).

Oryzalexin S is a phytoalexin against the devastating fungal blast pathogen *Magnaporthe oryzae*, and is derived from *syn*-stemar-13-ene via hydroxylation at C2α and C19 (Tamogani et al. 1993). The relevant OsKSL8, acting on the *syn*-CPP produced by OsCPS4, has been identified, with the encoding gene found on chromosome 11 (Nemoto et al. 2004). In addition, a closely related (both mechanistically and phylogenetically) *syn*-stemod-13(17)-ene synthase was then identified (Morrone et al. 2006). Although the relevant gene loci was not found in the available genome sequence, the relatively low sequence identity (< 93% nucleotide sequence identity) suggested that it was not an allele of *OsKSL8*, as other KSL alleles (even with functionally distinct activity and from different ssp.) shared > 99% identity (Xu et al. 2007), and so this was termed *OsKSL11*. However, it has since been clarified that *OsKSL11* is a distinct allele of *OsKSL8* derived from ssp. indica rice, which can then be termed *OsKSL8i*, while the other ssp. japonica derived allele is then *OsKSL8j* (Toyomasu et al. 2016).

Here the CYPs catalyzing hydroxylation at C19 and, subsequently, C2α of *syn*-stemarene to generate oryzalexin S were identified as the closely related CYP99A2/3 from the *c4BGC* and CYP71Z21/22 from the *c7BGC*, respectively. Intriguingly, utilizing the recently reported phylogenetically representative set of pan-rice genomes (Zhou et al. 2020), it was found that the relevant *syn*-stemarene producing *OsKSL8j* is actually not present in the examined (sub)tropical spp. japonica cultivars, having been replaced by the functionally distinct, *syn*-stemodene producing *OsKSL8i* (*OsKSL11*), the implications of which are discussed.

## Materials and methods

Unless otherwise stated, all chemicals were obtained from Fisher Scientific. The genes utilized here were largely synthetic, optimized for expression in *Escherichia coli*, as previously reported—i.e., *sCYP99A2* and *sCYP99A3* (Wang et al. 2011), as well as *sCYP71Z6* and *sCYP71Z7* (Wu et al. 2011), and *sCYP71Z22*, but the native *CYP71Z21* (Liang et al. 2021). These CYPs were expressed as described (Kitaoka et al. 2015), either with just the CYP reductase (CPR) from *Arabidopsis thaliana* (AtCPR1) for feeding studies, or in a previously described modular metabolic engineering system (Cyr et al. 2007), as adapted for CYP expression (Kitaoka et al. 2015), with expression of a GGPP synthase (*AgGGPS*), *OsCPS4* and *sOsKSL8j* to produce *syn*-stemar-13-ene. As needed, metabolic flux was increased as previously described (Morrone et al. 2010). The resulting diterpenoids were extracted and analyzed by GC–MS and, as necessary, purified by flash chromatography and HPLC, using GC–MS analysis to track the targeted compounds, which were then structurally analyzed by NMR and/or utilized for feeding studies, all as has been previously described—e.g., (Swaminathan et al. 2009; Wang et al. 2011, 2012; Wu et al. 2011, 2013; Kitaoka et al. 2015). Gene presence was determined via BLAST searches of the relevant genes from cv. Nipponbare (ssp. japonica) against the cDNA databases for the described (sub)species and, where relevant, cultivars at http://oryza.gramene.org (Tello-Ruiz et al. 2022). Phylogenetic analysis of *KSL8* was carried out using CLC Main WorkBench 22 (Qiagen Aarhus A/S).

## Results

**Fig. 1:**
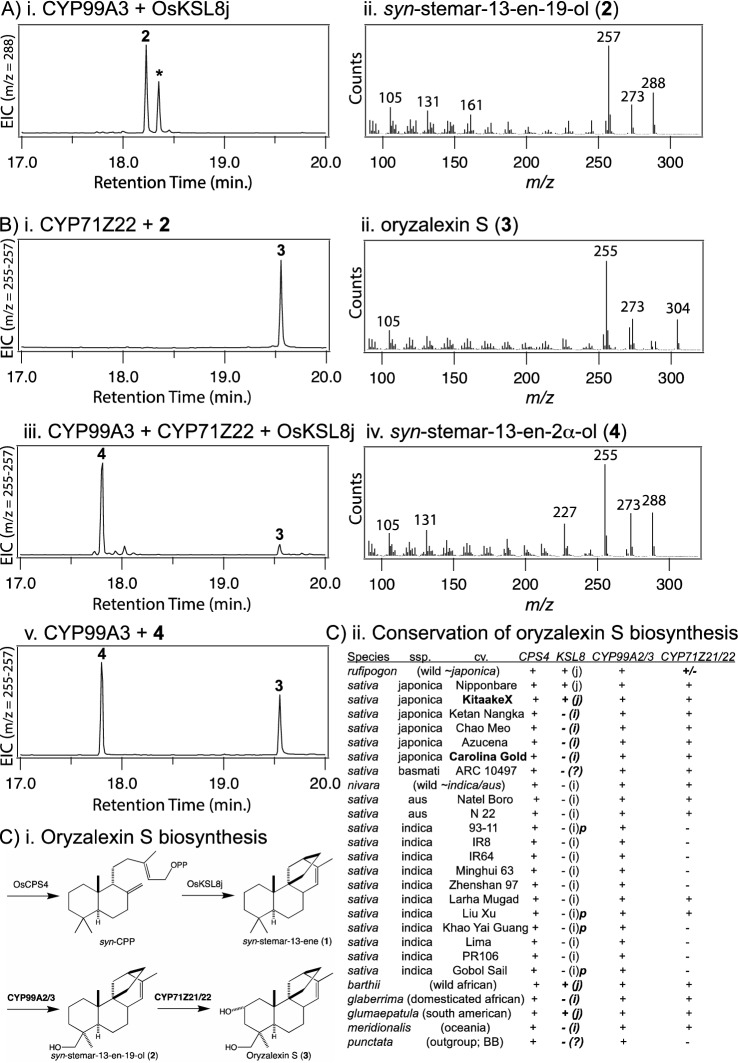
**A** CYP99A2/3 catalyze 19-hydroxylation of *syn*-stemar-13-ene (**1**). i) Extracted ion count (EIC) chromatogram from GC–MS analysis of extracts from *E. coli* co-expressing CYP99A3 and the requisite AtCPR1 as well as engineered to produce **1** via co-expression of a GGPP synthase and OsKSL8j. Peak marked with * is presumed to be 19-hydroxy derivative of *syn*-stemod-12-ene, which is produced in approximately the same ratio relative to **1** by OsKSL8j as seen here for these hydroxylated products (Xu et al. 2007). ii) Mass spectra of *syn*-stemar-13-en-19-ol (**2**). See Supplemental Fig. S1 for verification of CYP99A2 activity and additional GC–MS data supporting assignment of * as *syn*-stemod-12-en-19-ol. **B** CYP71Z21/22 catalyze 2α-hydroxylation. i) EIC chromatogram from GC–MS analysis of extracts from *E. coli* co-expressing CYP71Z22 and the requisite AtCPR1, and then fed *syn*-stemar-13-en-19-ol (**2**). See Supplemental Fig. S2 for verification of CYP71Z21 activity. ii) Mass spectra of oryzalexin S (**3**). iii) EIC chromatogram from GC–MS analysis of extracts from *E. coli* co-expressing CYP99A3, CYP71Z21 and the requisite AtCPR1 as well as engineered to produce - **1**. iv) Mass spectra of *syn*-stemar-13-en-2α-ol (**4**). v) EIC chromatogram from GC–MS analysis of extracts from *E. coli* co-expressing CYP99A3 and the requisite AtCPR1, and then fed *syn*-stemar-13-en-2α-ol (**4**) purified from *E. coli* co-expressing CYP71Z22 and the requisite AtCPR1 as well as engineered to produce **1** (see Supplemental Fig. S3 for EIC chromatogram from GC–MS analysis of extract). **C** Conservation of Oryzalexin S. i) Biosynthetic pathway, with previously identified diterpene cyclases on top and CYPs identified here below (also indicated by bold text). ii) Conservation of relevant genes (bold italic text indicates those explicitly discussed here)

Based on the previously reported ability of CYP99A3 to catalyze at least C19-hydroxylation of *syn*-stemodene (Wang et al. 2011), the activity of both this and the closely related paralog CYP99A2 with the oryzalexin S precursor *syn*-stemar-13-ene (**1**) was examined. Specifically, these CYPs were each co-expressed with the requisite CPR in *E. coli* also engineered to produce this putative substrate. In both cases this led to observation of what appeared to be hydroxylated derivatives of **1**, along with the accompanying *syn*-stemod-12-ene also made by OsKSL8j in approximately the same ratio relative to **1** as seen here for these hydroxylated products (Xu et al. 2007), as indicated by the increase in molecular weight (MW) from 272 to 288 Da (Fig. 1Ai, ii and Supplemental Fig. S1). For structural analysis, metabolic flux to isoprenoids was increased and the culture volume increased with CYP99A3 (as this seems to be more efficient than CYP99A2, see Supplemental Fig. S1), enabling purification of sufficient amounts of this diterpenoid for NMR analysis (Supplemental Table S1), which revealed the major product to be the expected C19-hydroxylated derivative *syn*-stemar-13-en-19-ol (**2**).

Previous work indicated that CYP71Z6 and CYP71Z7 from the *c2BGC* act on C2 of specific (*ent*-CPP derived) labdane-related diterpenes (Wu et al. 2011), producing C2α-hydroxy derivatives (Kitaoka et al. 2015). Accordingly, neither was found to modify *syn*-stemarene. Here, feeding experiments demonstrated these also do not react with *syn*-stemar-13-en-19-ol (data not shown). Given the recent discovery of roles for the CYP71Z sub-family members from the *c7BGC* in diterpenoid phytoalexin biosynthesis, despite their activity with the macrocyclic *ent*-casbene, which is not a labdane-related diterpene as it is derived from direct cyclization of GGPP by a class I diterpene synthase, these also were investigated. Fortuitously, feeding experiments found that, while CYP71Z2 was unreactive, the closely related CYP71Z21 and CYP71Z22 both efficiently converted *syn*-stemar-13-en-19-ol to oryzalexin S (Fig. 1Bi, ii and Supplemental Fig. S2). This demonstrates CYP71Z21/22 catalyze the necessary C2α-hydroxylation. In addition, co-expression of CYP71Z22 and CYP99A3 (as well as AtCPR1) in *E. coli* also engineered to produce *syn*-stemarene led to production of oryzalexin S (**3**), along with a substantial amount of an alternative hydroxylated derivative (Fig. 1Biii, iv). To verify this product was the expected *syn*-stemar-13-en-2α-ol (**4**), CYP71Z22 was utilized in the metabolic engineering system (Supplemental Figure S3), along with increased metabolic flux to isoprenoids and larger culture volume, enabling purification of sufficient amounts of this diterpenoid to feed to CYP99A3, which was able to convert this to oryzalexin S (Fig. 1Bv). The less efficient production of oryzalexin S (**3**) from *syn*-stemar-13-en-2α-ol (**4**) by CYP99A3, relative to *syn*-stemar-13-en-19-ol (**2**) by CYP71Z22, along with accumulation of **4** in the full-pathway containing *E. coli*, suggests preferential hydroxylation, with initial C19-hydroxylation of *syn*-stemarene by CYP99A2/3 and subsequent C2α-hydroxylation by CYP71Z21/22 in oryzalexin S biosynthesis (Fig. 1Ci).

Given the previously reported ssp. specific occurrence of both *OsKSL8j* and the *c7BGC*, especially the variation observed for the associated CYP71Z sub-family members, it seemed prudent to investigate distribution of the genes associated with oryzalexin S biosynthesis. For this purpose, the recently reported phylogenetically representative set of Asian rice genome sequences (Zhou et al. 2020), along with a broader set of domesticated and wild rice relatives across the *Oryza* genus, up to and including *Oryza punctata*, which served as an outgroup (Stein et al. 2018), were examined here (Fig. 1Cii). As previously reported, the *c4BGC* is widely conserved (Miyamoto et al. 2016), with *OsCPS4* and *CYP99A2/3* present in all these genomes, and the *c7BGC* is found in all ssp. japonica, although at least *CYP71Z21* is more widely distributed, including other species (Zhan et al. 2020). While the *O. rufipogon* landrace examined here (W1943) does not contain *CYP71Z21/22*, which clarifies the basis for the previously reported absence of oryzalexin S therein (Toyomasu et al. 2016), it was previously reported that 12/13 *O. rufipogon* landraces contain at least *CYP71Z21* (Zhan et al. 2020), hence the “±” indication at this point. More interestingly, substantial variation is observed with *KSL8*. Note that while the originally identified *OsKSL8j* was cloned from cv. Nipponbare, only a partial cDNA was found in the database examined here. Thus, while apparently partial cDNA are found for a few other *O. sativa* cultivars in this database (as indicated by appended “p”), these *OsKSL8* may be functional and were included in the phylogenetic analysis, enabling assignment of relevant allele. Despite association of the *syn*-stemarene producing allele with ssp. japonica, the corresponding *OsKSL8j* is only found in the temperate representative cv. Nipponbare, as well as the wild-rice relative *O. rufipogon*. The other (tropical and subtropical) representatives contain alleles phylogenetically closer to *OsKSL8i* (*OsKSL11*), with full-length open-reading frame (ORF) sequence identities > 95.5% versus < 92.5%, respectively. Consistent with retention in only the temperate cultivars of ssp. japonica, *OsKSL8j* was found in the temperate cv. KitaakeX (Jain et al. 2019), while *OsKSL8i* is found in the tropical cv. Carolina Gold (Vaughn et al. 2021). OsKSL8 from the more distant species of rice were included in this phylogenetic analysis (Supplemental Figure S4), allowing tentative assignment of allele in almost all the AA-genome rice, although not the more distant (BB-genome) *O. punctata*, in which the most closely related cDNA shared 85–88% ORF sequence identity with the other full-length *KSL8* examined here. Strikingly, the only other exception is found in the *O. sativa* ssp. basmati representative, where the most closely related cDNA shared < 82% ORF sequence identity with all other *KSL8* examined here (including that from *O. punctata*).

## Discussion

Here the CYPs catalyzing hydroxylation of *syn*-stemarene at C19 and, as shown here, the subsequent hydroxylation at C2α required for oryzalexin S biosynthesis were identified as the closely related paralogous pairs CYP99A2/3 and CYP71Z21/22 (respectively). The activity of CYP71Z21/22 is somewhat surprising given the differences between the macrocyclic *ent*-casbene that these react upon for *ent*-10-oxodepressin production versus the labdane-related (multicyclic) *syn*-stemar-13-en-19-ol these react with for oryzalexin S biosynthesis. Regardless, the encoding pair of genes are found in the previously identified *c4BGC* and *c7BGC* (respectively), with the former also containing that for the *syn*-CPP synthase *OsCPS4*, which is required for production of oryzalexin S (Zhang et al. 2021). By contrast, the gene for the relevant *syn*-stemarene synthase *OsKSL8(j*) is located elsewhere (chromosome 11), and then cross-stitches together these two *BGCs* to form the oryzalexin S biosynthetic pathway.

Given both *OsKSL8j* and the *c7BGC* are specifically associated with ssp. japonica (Toyomasu et al. 2016; Zhan et al. 2020), and, especially, the discovery that *OsKSL11* is a functionally distinct ssp. indica derived allele (more accurately termed *OsKSL8i*), a wider examination of the genes relevant to oryzalexin S biosynthesis was carried out here. The results were consistent with the previous suggestion that such metabolism was present in the progenitor of ssp. japonica and *O. rufipogon* (Toyomasu et al. 2016), with possibly more ancient origins suggested by the presence of the relevant genes more widely in the *Oryza* genus (although this latter hypothesis needs further investigation). Strikingly, in *O. sativa* ssp. japonica it appears the *OsKSL8j* allele has only been retained in temperate cultivars, with those from (sub)tropical climates containing *OsKSL8i* (*OsKSL11*) instead. While gene flow via hybridization and introgression from ssp. japonica into ssp. indica is well known (Wing et al. 2018), these results indicate introgression of this functionally distinct allele in the opposite direction (i.e., from ssp. indica into ssp. japonica), with the accompanying disappearance of oryzalexin S. Indeed, an even more extreme case is found in ssp. basmati, where a highly divergent allele appears. Thus, it will be of interest to determine the product of this OsKSL8 variant. In addition, although CYP99A3 from the widespread c4BGC has been reported to catalyze oxygenation at C19 of the *syn*-stemod-13(17)-ene product of OsKSL8i (OsKSL11) (Wang et al. 2011), this is not sufficient for diterpenoid solubility and bioactivity, which invariably requires at least two spatially separated oxy groups (Wu et al. 2013). However, *syn*-stemod-13(17)-en-19-ol is not further transformed by CYP71Z21/22 (data not shown). Accordingly, no *syn*-stemod-13(17)-ene derived phytoalexin has yet been identified, so it will be of interest to investigate any additional oxygenation of this to examine bioactivity and determine the basis for introgression of *OsKSL8i* into (sub)tropical ssp. japonica, as well as the more divergent allele found in ssp. basmati.

## Supplementary Information

Below is the link to the electronic supplementary material.

Supplementary file1 (PDF 242 KB)

## Data Availability

All data generated or analyzed during this study are available from the corresponding author upon reasonable request.
