# Supplementary material for: Oryzalexin S biosynthesis: a cross-stitched disappearing pathway
Source: aBIOTECH. 2023 Jan 19;4(1):1–7. doi: 10.1007/s42994-022-00092-3 (PMC10199973; doi:10.1007/s42994-022-00092-3)
Supplement: Supplementary file 1 — Supplementary file1 (PDF 242 KB) [file 42994_2022_92_MOESM1_ESM.pdf]

<u>Supplemental Data for</u>:

Oryzalexin S biosynthesis: A cross-stitched disappearing pathway

Le Zhao†, Richard Oyagbenro†, Yiling Feng, Meimei Xu and Reuben J. Peters*

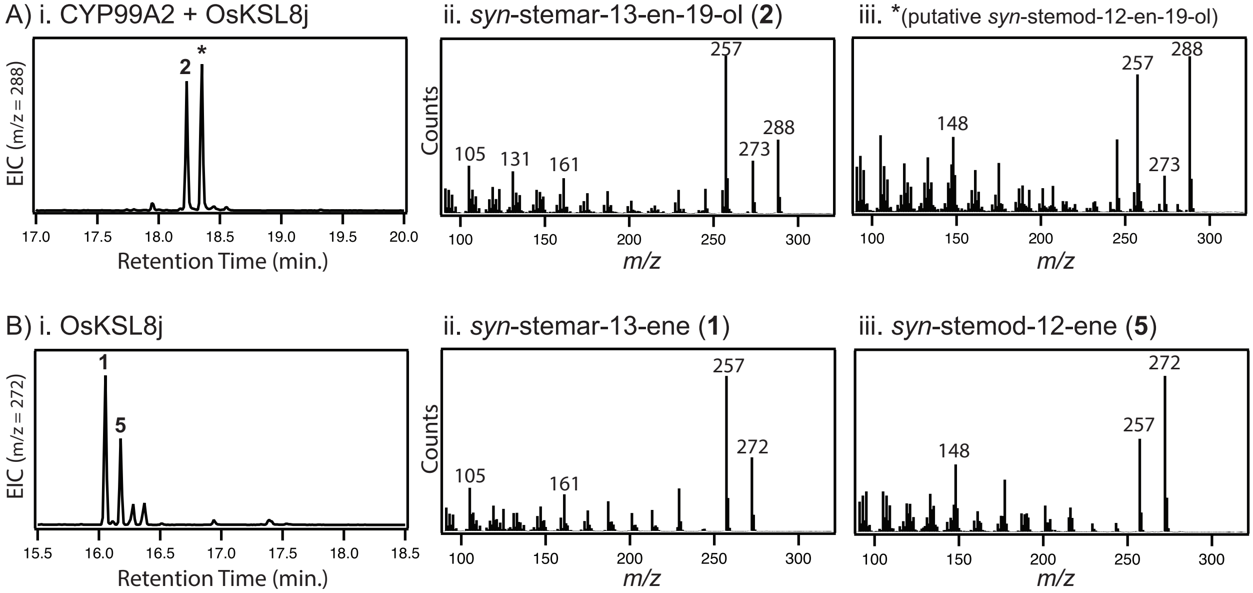

Figure S1: CYP99A2 activity and comparison of observed products with the two major olefins produced by OsKSL8j. A) GC-MS analysis of extracts from *E. coli* co-expressing CYP99A3 and the requisite AtCPR1 as well as a GGPP synthase and OsKSL8j. i) Extracted ion count (EIC) chromatogram. ii) Mass spectra of *syn*-stemar-13-en-19-ol (**2**). iii) Mass spectra of peak marked with *. This is putatively assigned as *syn*-stemod-12-en-19-ol based on comparison of relative retention time and mass spectra with the *syn*-stemod-12-ene (**5**) co-product of OsKSL8j. Note that CYP99A2 seems to produce this more efficiently than **2** relative to CYP99A3 (c.f., Figures 1Ai and S1Ai). B) GC-MS analysis of extracts from *E. coli* co-expressing a GGPP synthase and OsKSL8j. i) EIC chromatogram. ii) Mass spectra of *syn*-stemar-13-ene (**1**), precursor of **2**. iii) Mass spectra of *syn*-stemod-12-ene (**5**), putative precursor of * product.

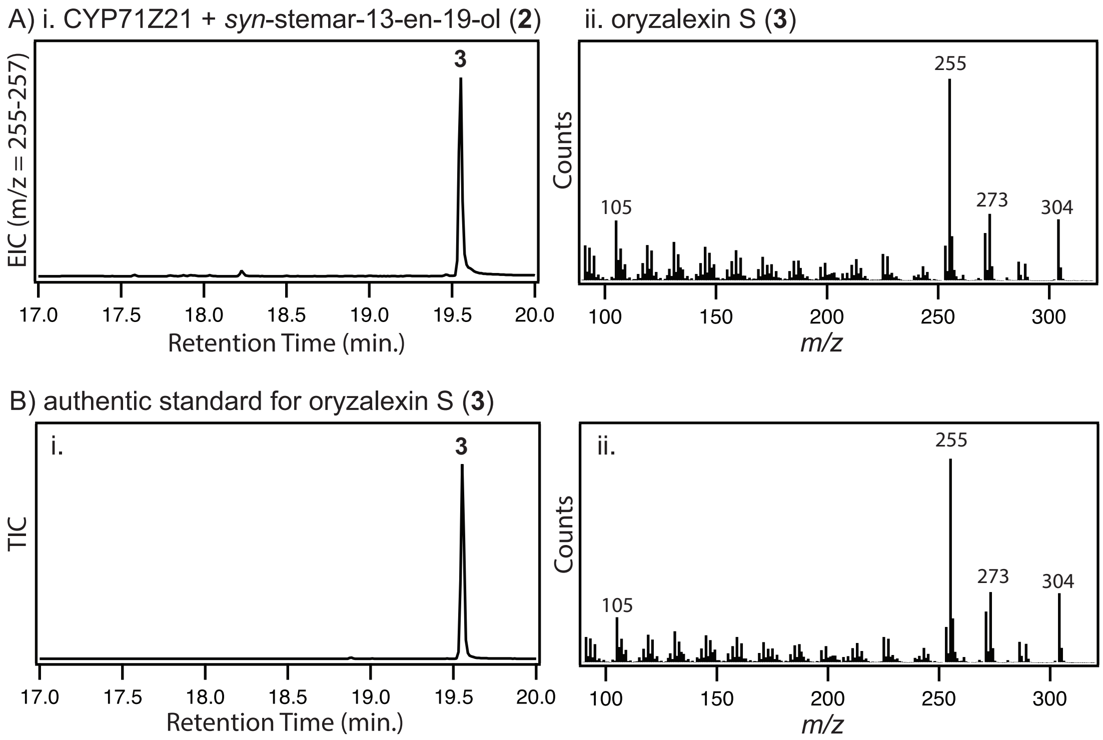

Figure S2: CYP71Z21 activity with *syn*-stemar-13-en-19-ol (**2**) and comparison of observed product with an authentic standard for oryzalexin S (**3**). Note that neither of CYP71Z21/22 react with the putative *syn*-stemod-12-en-19-ol (*). A) GC-MS analysis of extracts from *E. coli* co-expressing CYP71Z21 and the requisite AtCPR1, and then fed **2**. i) Extracted ion count (EIC) chromatogram. ii) Mass spectra of product (**3**). B) GC-MS analysis of authentic oryzalexin S (**3**). i) Total ion count (TIC) chromatogram. ii) Mass spectra.

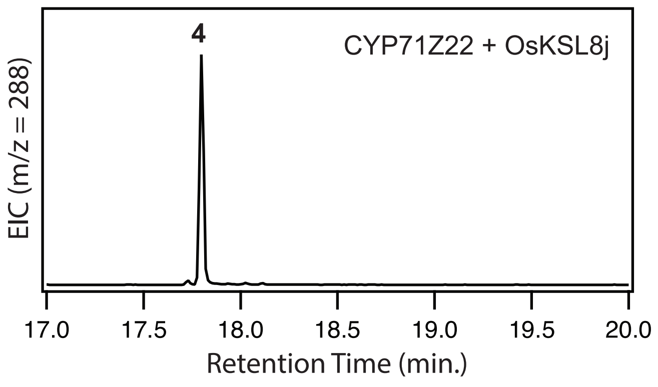

Figure S3: Extracted ion count (EIC) chromatogram from GC-MS analysis of extracts from *E. coli* co-expressing CYP71Z22 and the requisite AtCPR1 as well as a GGPP synthase and OsKSL8j. Note the lack of other products suggests CYP71Z22 is specific for *syn*-stemar-13-ene (**1**).

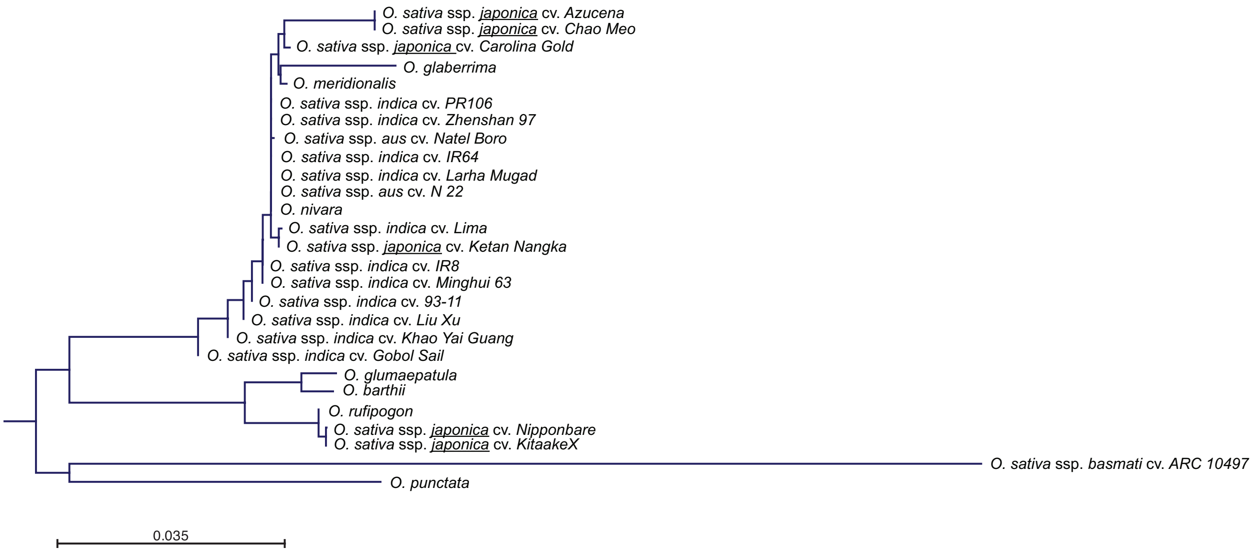

Figure S4: Phylogenetic tree for *KSL8* across the *Oryza* genus. Sequences were downloaded from the described BLAST search against the cDNA databases from Gramene-Oryza and truncated to the largest open-reading frame. These were then aligned (gap open cost = 10, gap extension cost = 1, gap end cost = free, and very accurate settings), and used as input to create tree (tree construction method = Neighbor Joining, nucleotide distance measure = Jukes-Cantor; bootstrap replicates = 1000). The *O. punctata* sequence was hypothesized to serve as the outgroup, but the distance observed for that from *O. sativa* ssp. *basmati* (cv. *ARC 10497*) strongly indicated this also is an outgroup. Thus, these were manually set as the outgroup to root the phylogram shown here. Sequences from ssp. japonica are highlighted by underlining.

Table S1: Chemical shift data for *syn*-stemar-13-en-19-ol (**2**).

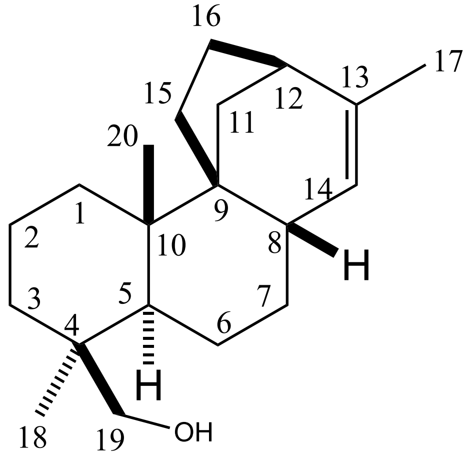

Position	δ_C (ppm)	δH (ppm), multiplicity, *J* (Hz)
1	29.8	1.56 (m), 1.39 (m)
2	18.2	1.48 (m), 1.39 (m)
3	35.3	0.83 (m), 1.76 (dq, *J*=13.6, 2.8)
4	38.5	
5	50.0	1.18 (m)
6	21.9	1.55 (m), 1.25 (m)
7	31.7	1.09 (m), 1.19 (m)
8	44.1	1.9 (d, *J*=12.5)
9	50.9	
10	38.6	
11	31.8	1.32 (m), 1.62 (m)
12	43.1	2.13 (t, *J*=4.6)
13	138.6	
14	123.7	4.87 (m)
15	32.5	1.41 (m), 1.13 (m)
16	32.2	1.42 (m), 1.46 (m)
17	26.9	0.88 (s)
18	22.0	1.56 (s)
19	65.3	3.34 (dd, *J*=10.8, 1.3), 3.73 (d, *J*=10.8)
20	17.7	0.86 (s)
